# Nearest-neighbor amino acids of specificity-determining residues influence the activity of engineered Cre-type recombinases

**DOI:** 10.1038/s41598-020-70867-5

**Published:** 2020-08-19

**Authors:** Anjali Soni, Martina Augsburg, Frank Buchholz, M. Teresa Pisabarro

**Affiliations:** 1 Structural Bioinformatics, BIOTEC, TU Dresden, Tatzberg 47-51, 01307 Dresden, Germany; 2 University Carl Gustav Carus and Medical Faculty, UCC, Medical Systems Biology, TU Dresden, Fetscherstrasse 74, Dresden, Germany

**Keywords:** Computational models, Protein design, Protein function predictions, Molecular engineering

## Abstract

The tyrosine-type site-specific DNA recombinase Cre recombines its target site, *loxP*, with high activity and specificity without cross-recombining the target sites of highly related recombinases. Understanding how Cre achieves this precision is key to be able to rationally engineer site-specific recombinases (SSRs) for genome editing applications. Previous work has revealed key residues for target site selectivity in the Cre/*loxP* and the related Dre/*rox* recombinase systems. However, enzymes in which these residues were changed to the respective counterpart only showed weak activity on the foreign target site. Here, we use molecular modeling and dynamics simulation techniques to comprehensively explore the mechanisms by which these residues determine target recognition in the context of their flanking regions in the protein–DNA interface, and we establish a structure-based rationale for the design of improved recombination activities. Our theoretical models reveal that nearest-neighbors to the specificity-determining residues are important players for enhancing SSR activity on the foreign target site. Based on the established rationale, we design new Cre variants with improved *rox* recombination activities, which we validate experimentally. Our work provides new insights into the target recognition mechanisms of Cre-like recombinases and represents an important step towards the rational design of SSRs for applied genome engineering.

## Introduction

**Figure 1:**
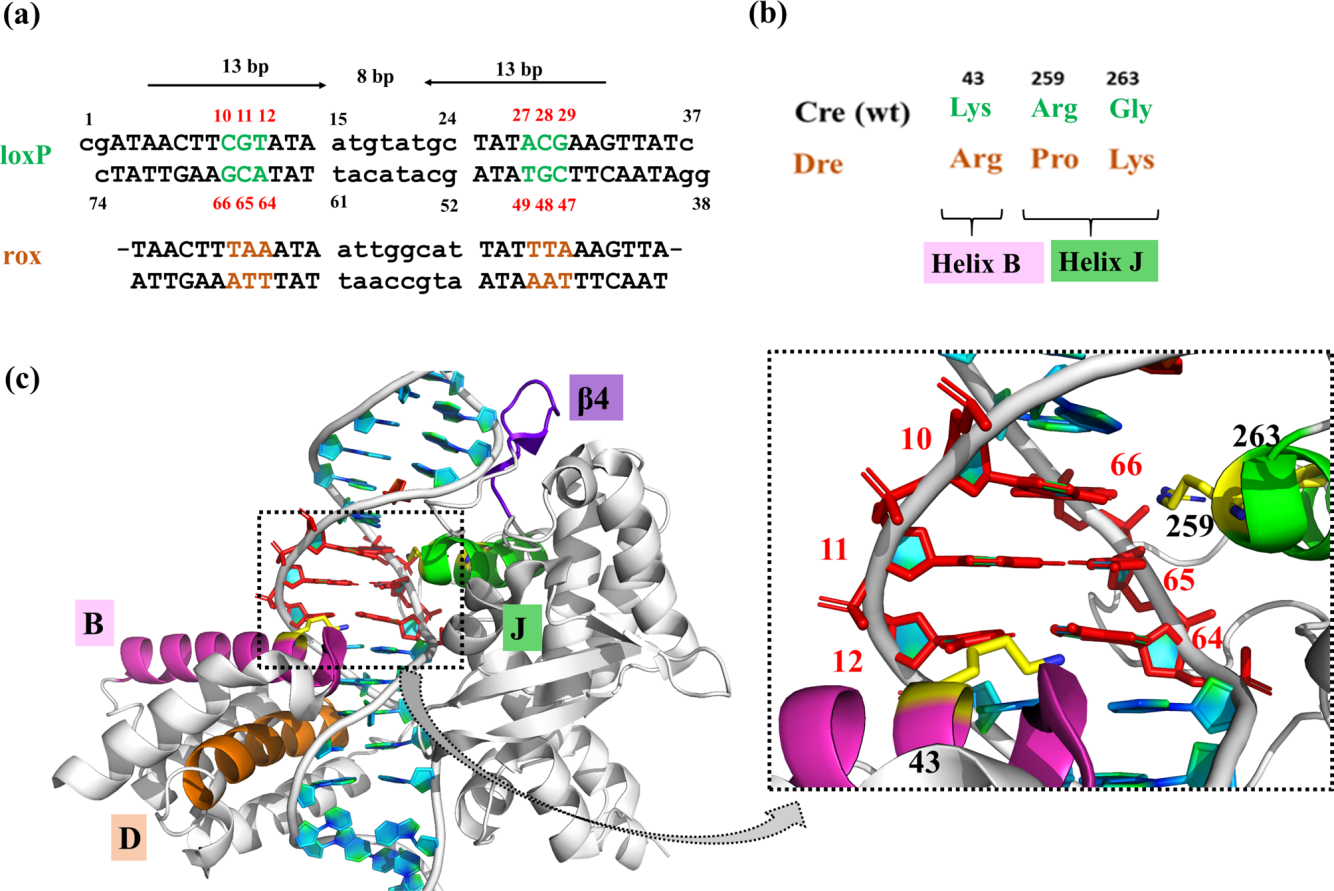
Nucleotides and amino acids at the protein-DNA interface of Cre/*loxP* and Dre/*rox* recombinase systems. (**a**) Sequences of *loxP* and *rox* target sites. The three nucleotides that differ between *loxP* and *rox* are highlighted in color. The numbering of the individual bases is provided for the upper and lower DNA strand, respectively. (**b**) Protein amino acids facing the altered DNA bases of *loxP* and *rox*. (**c**) Snapshot of the cleaving monomer of the Cre/*loxP* complex (PDB 1Q3U). The recognition regions at the protein-DNA interface are displayed in magenta (helix B), orange (helix D), green (helix J) and purple (beta 4). The area of interest in this study (*i.e.* PDI_BJ_ area; the protein-DNA interface formed by amino acids of helix B and J and nucleotides 10/66, 11/65, and 12/64) is zoomed in, and relevant bases and amino acids are labeled. The numbering used is based on the Cre/*loxP* crystal structure (for details see Fig. S1). Figure generated using Pymol (version 2.1, https://pymol.org/).

Site-specific DNA recombinases (SSRs) are powerful tools for precise DNA rearrangements to allow inversions, deletions and translocations in the genome of heterologous hosts^1–4^. The Cre/*loxP* recombinase system is a well-validated and extensively studied member of the tyrosine SSRs protein family^5,6^. The Cre enzyme (Causes recombination) from bacteriophage P1 is recognized as a prevalent tool for genetic alterations due to its efficiency and specificity to recombine its native DNA target sequence (*loxP*) and because of its simplicity of use, i.e. no accessory proteins are required for recombination catalysis^5,7^. Cre specifically recombines *loxP*, which is composed of two 13 base pair (bp) palindromic sequences parted by a spacer region of 8 bp (Fig. 1a)^8^. Each half-site of *loxP* is recognized by one Cre monomer. Cre-mediated recombination requires the formation of a synapse comprising a Cre tetramer recognizing two *loxP* sites. To start the recombination reaction, two Cre monomers in the tetrameric complex initiate the cleavage, whereas the other two are in an inactive or non-cleaving conformation^1,5^. The multi-step recombination event proceeds through the formation of a Holliday junction intermediate undergoing isomerization between the cleaving and non-cleaving conformations to complete reaction catalysis^4^.

SSRs have become indispensable for complex DNA manipulation due to their precise and unique ability to rearrange DNA both in vitro and in vivo, supporting many applications in biomedicine and biotechnology. To extend the utility of SSRs, recent efforts have focused on finding additional naturally occurring Cre-like SSRs and their respective target sites. The discovery of several such systems, including the Dre/*rox*^9^, VCre/*VloxP*^10^, SCre/*SloxP*^10^, Vika/*vox*^11^, Nigri/*nox*^12^ and Panto/pox^12^ recombinase systems has greatly expanded the repertoire of available SSRs that can be used alone or in combination to allow advanced genome engineering^13–15^, and to build sophisticated synthetic biology circuits^16,17^. Importantly, while these enzymes, as well as their target sites, share high sequence similarities, cross-recombination is typically not observed. For instance, Cre shares 41% sequence identity with Dre, and their respective DNA target sites only differ in 3 out of the 13 bp per half-site (Fig. 1a). Nevertheless, Cre does not show activity on *rox,* and, similarly, Dre is inactive on *loxP*^18^. In previous work, detailed comparative analyses of these recombinases have identified the amino acids K43, R259, and G263 of Cre as critical residues for the discrimination between the *loxP* and *rox* sites^12^. Indeed, the substitution of these three amino acids in Cre by the corresponding Dre residues (mCre_K_ variant: K43R, R259P, and G263K) (Fig. 1b) was sufficient to confer selectivity for *rox*, albeit at low activity. In order to establish a structure–function rationale, which could help in guiding further efforts for improving recombination properties, we decided to investigate in detail the molecular recognition mechanisms of these specificity-determinant key residues at positions 43, 259 and 263 in binding to *loxP* and *rox* sites.

## Results and discussion

Based on the crystal structure of Cre bound to *loxP* in the synaptic state (PDB 1Q3U^19^), we generated three-dimensional (3D) molecular models of Dre/*rox* and mCre_K_ in complex with *loxP* and *rox* target sites. Utilizing structure-based modeling and molecular dynamics (MD) simulations, we explored the interactions involved in protein-DNA binding in these complexes, particularly at the interface formed by the specificity-determining amino acids at positions 43, 259 and 263, which lay on helix B and J, and the nucleotides at positions 10/66, 11/65, and 12/64 (Fig. 1b,c). Hereafter, we named this interfacial region as the PDI_BJ_ area.

### MD-based recognition analysis of Cre/*loxP*

**Figure 2:**
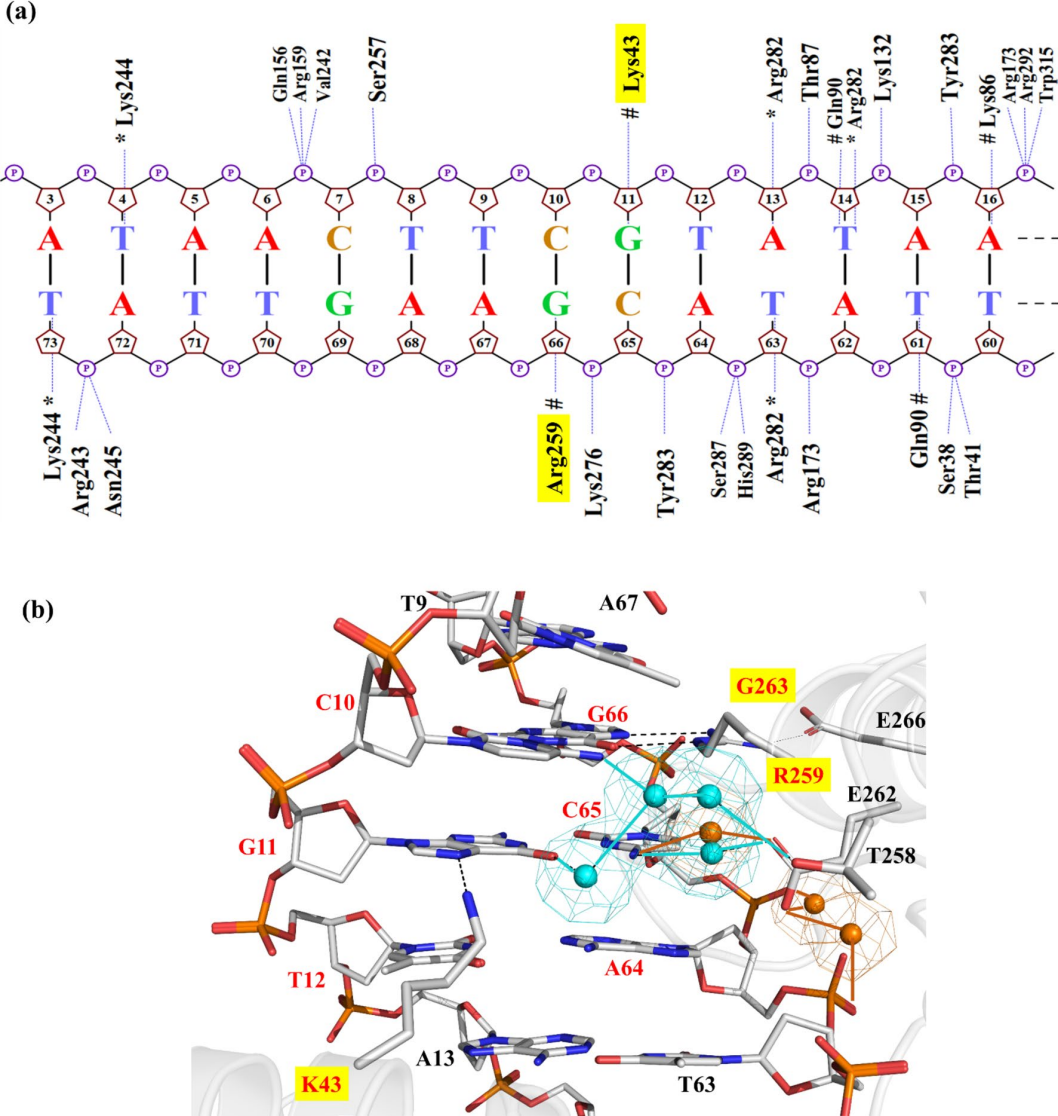
Analysis of protein-DNA interactions in Cre/*loxP* (**a**) Projection of hydrogen bond interactions spotted in the last frame of the Cre/*loxP* MD trajectory. The base-specific interactions with the minor groove are labeled with an asterisk (*). Hashes (#) denote the interactions with the major groove. Nucplot was used to generate this image. (**b**) Detailed view of interactions observed in the Cre/*loxP* crystal structure and MD simulation in the PDI_BJ_ area. Hydrogen bonds are shown in black dashed lines. The specificity-determining residues are highlighted with a yellow box. Water positions predicted by WaterMap (version 1.0, https://www.schrodinger.com/) are shown in orange spheres, and water-mediated contacts are depicted by orange lines. For comparison, a network of water molecules found in this area in another Cre/*loxP* crystal structure (PDB 3C29) is shown superimposed with the Cre/*loxP* MD-refined structure (cyan spheres and lines). Pymol was used to generate the image (version 2.1, https://pymol.org/).

In order to better understand the molecular recognition mechanisms of the mCre_K_ variant containing mutations at positions 43, 259 and 263 with respect to *wild type* Cre, we decided to first perform a comprehensive comparative analysis of the recognition properties of the wild type Cre/*loxP* and Dre/*rox* recombinase systems. For this purpose, we chose available structural information on the Cre/*loxP* synaptic complex obtained by X-ray crystallography (see “Materials and methods” for details) and performed MD simulations to examine at atomic detail the protein-DNA recognition and its binding energetics. Results obtained from the MD simulations underlined the crucial interactions prevalent in the Cre/*loxP* complex in the cleaving and non-cleaving conformers. The interactions observed for the cleaving conformer are shown in Figs. 2a and S1. The protein-DNA hydrogen bonds observed in our simulations are in agreement with those observed in the Cre/*loxP* crystal structure. Table S1 provides a comprehensive description of the hydrogen bonds observed in the MD trajectory based on their frequency of occurrence. At the investigated PDI_BJ_ area (*i.e.* the interface formed by the specificity-determining amino acids at positions 43, 259, 263 of helix B and J, and bases at positions 10/66, 11/65, and 12/64, Fig. 1c), the residues K43, R259 and G263 recognize the central DNA base pairs C10/G66, G11/C65, T12/A64, and A13/T63 through direct hydrogen bonding (Fig. 2a). Residue K43 acts as a hydrogen-bond donor interacting with G11(N7 atom) in the major groove of the DNA (appearing in 73% of the simulation time, Fig. 2b and Table S1). Lysine may adopt different accessible rotamers in folded states of proteins^20^. As such, K43 can be found interacting dynamically with different bases in different crystal structures of Cre/*loxP* (i.e. with T63(O4) in PDB 1Q3U, and with G11(N7) in PDB 3C29). Our MD simulation predominantly supports the interaction with G11(N7). Residue R259 forms a strong bidentate hydrogen bond with the *loxP* bases G66(N7) (55% of the MD trajectory) and G66(O6) (40% of the MD trajectory) (Table S1). This interaction is crucial for Cre/*loxP* recognition^21^. Position 259 is particularly interesting from the recognition perspective in SSRs, as it can accommodate a variety of mutations to maintain specific contacts of different physico-chemical nature with the bases of the DNA target site^22,23^. Residue G263 provides stability to helix J by allowing E266 to interact with R259 (through its free NH1 atom) and therefore applying constraints to its orientation (Fig. 2b).

Because water molecules can be crucial in defining the structure, function and stability of protein-DNA complexes^24–26^, we also analyzed water-mediated contacts in the Cre/*loxP* complex using WaterMap^27^. The examination of the interfacial solvent indicated the presence of a water molecule in the major groove bridging the protein residue E262 to the base C65 (Fig. 2b). Interestingly, this predicted water-mediated contact coincides with an equivalent observed in another synaptic crystal structure of Cre/*loxP* (PDB 3C29^28^; Fig. 2b). Residue E262 is regarded as “guardian for *loxP* selectivity” as it is responsible for modulating DNA binding and discriminating *loxP* from other substrates^4,29^. We also observed a water-mediated protein–DNA interaction between the carboxylate group of E262 and the phosphates of the bases C65 and A64, which could point towards another preferential water-mediated contact not observed in the available crystallographic data, probably due to resolution restrictions. In view of the ion distribution in the Cre/*loxP* complex, a high density of K^+^ is observed in the DNA major groove of the investigated PDI_BJ_ area, which could be linked to the presence of a GC-rich region, and in a lower extent in the *loxP* minor groove^30,31^ (Fig. S2a). Ions were also observed accumulating at the entrance of the major groove, as their presence will minimize the repulsion between residue E262 and DNA phosphates (see spatial location of E262 on helix J in Fig. 2b).

### MD-based recognition analysis of Dre/*rox*

To perform a comprehensive comparative analysis, we investigated in detail the protein-DNA recognition properties in the Dre/*rox* recombinase system. For this purpose, we built a 3D molecular model of Dre in complex with *rox* by using available structural data at the Protein Data Bank (PDB^32^) on the Cre/*loxP* system as template and different software tools as validation for our modeling (see “Materials and methods” and Supplementary Information for details). The comparison of the resulting 3D Dre/*rox* models obtained by different means (i.e. MODELLER/Discovery Studio (DS)^33,34^ and the SWISS-MODEL^35^ and PHYRE2^36^ webservers) with respect to the Cre/*loxP* structure showed similar RMSD’s values and, therefore structural agreement substantiating our modeling (*i.e.* heavy-atom RMSD of 1.59, 1.56 and 1.60 Å respectively, Fig. S3). For further studies, we chose the model obtained from DS.

**Figure 3:**
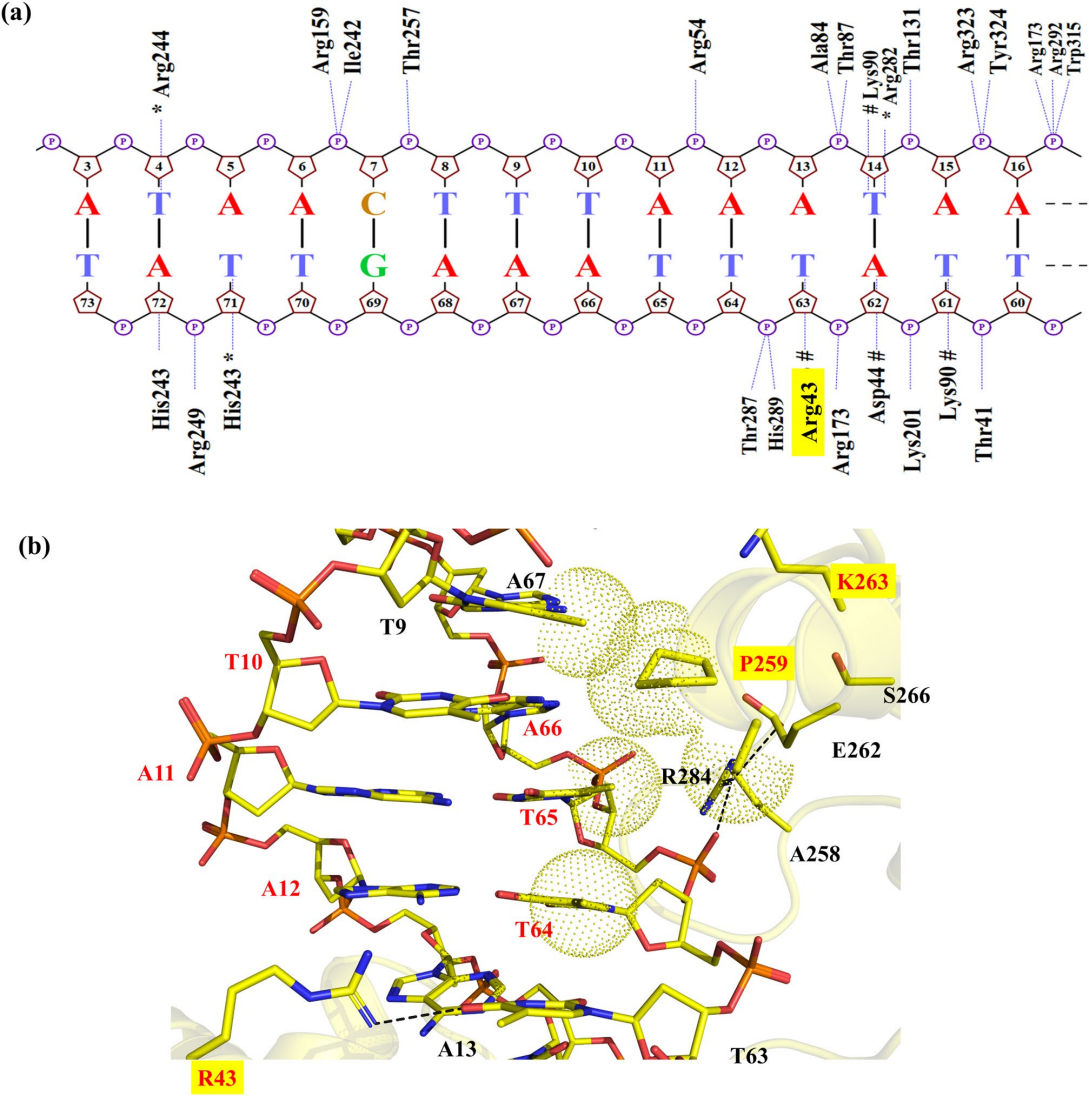
Analysis of protein-DNA interactions observed in the MD simulation of Dre/*rox* (**a**) Projection of hydrogen bond interactions spotted in the last frame of the Dre/*rox* MD trajectory. The base-specific interactions with the minor groove are labeled with an asterisk (*). Hashes (#) denote the interactions with the DNA major groove. Nucplot was used to generate the image. (**b**) Detailed view of interactions observed in the PDI_BJ_ area of the Dre/*rox* complex based on MD simulations. The specificity-determining residues are highlighted with a yellow box. Hydrogen bonds are shown in black dashed lines and van der Waals interactions in dotted spheres. Pymol was used to generate the image (version 2.1, https://pymol.org/).

The MD-based analysis of the obtained Dre/*rox* model indicated important amino acids involved in molecular recognition through hydrogen bond interactions with the DNA minor groove (H243, R244, R282) and major groove (R43, D44, K90) (Figs. 3a and S1). In comparison to Cre/*loxP*, the Dre/*rox* complex provides a modified interaction profile with less base-specific and more non-specific contacts (i.e. DNA backbone) (Figs. 3a and S1). A comprehensive description of the hydrogen bonds observed in the MD trajectory based on their frequency of occurrence is provided in Table S2.

In the Dre/*rox* complex, residues R43, P259 and K263 were predicted to recognize the three bp T10/A66, A11/T65, A12/T64 and A13/T63 through a combination of hydrogen bonds and van der Waals contacts (Fig. 3b). Residue R43 was found to act as a hydrogen-bond donor interacting with O4 of T63 (at ~ 70% and 14% frequency of occurrence for atoms R43(NH1) and R43(NH2), respectively) (Fig. 3b and Table S2). Residue P259 established hydrophobic contacts with the methyl groups of T9 and T65 consequently forming a well-packed interface at the major groove of *rox*. Alanine at position 258 was also found to be contributing partially to these hydrophobic contacts. Residue K263 did not interact with any particular amino acid or base. With the presence of Proline at position 259 and Serine at position 266, an intra-helical contact between these residues was not observed in the Dre/*rox* complex. In comparison, in the Cre/*loxP* complex, residue E266 locks the orientation of R259 by forming a hydrogen bond and thus contributing to stabilizing the helix (Figs. 2b, 3b). However, this stabilization of the helix in the Dre/*rox* complex is provided by R284 (L284 in Cre), which interacts through hydrogen bonds with E262 and with the phosphate of T65. Water-mediated interactions were not observed in the PDI_BJ_ area of the Dre/*rox* complex. Unlike Cre/loxP, the Dre/rox complex lacks the K^+^ density at the major groove of the DNA. As this complex also contains glutamic acid at position 262, the high K^+^ density is observed at the entrance of the major groove to minimize the repulsion between the respective amino acid and DNA phosphates (Fig. S2b).

### MD-based comparative recognition analyses of mCre_K_/*loxP* and mCre_K_/*rox*

**Figure 4:**
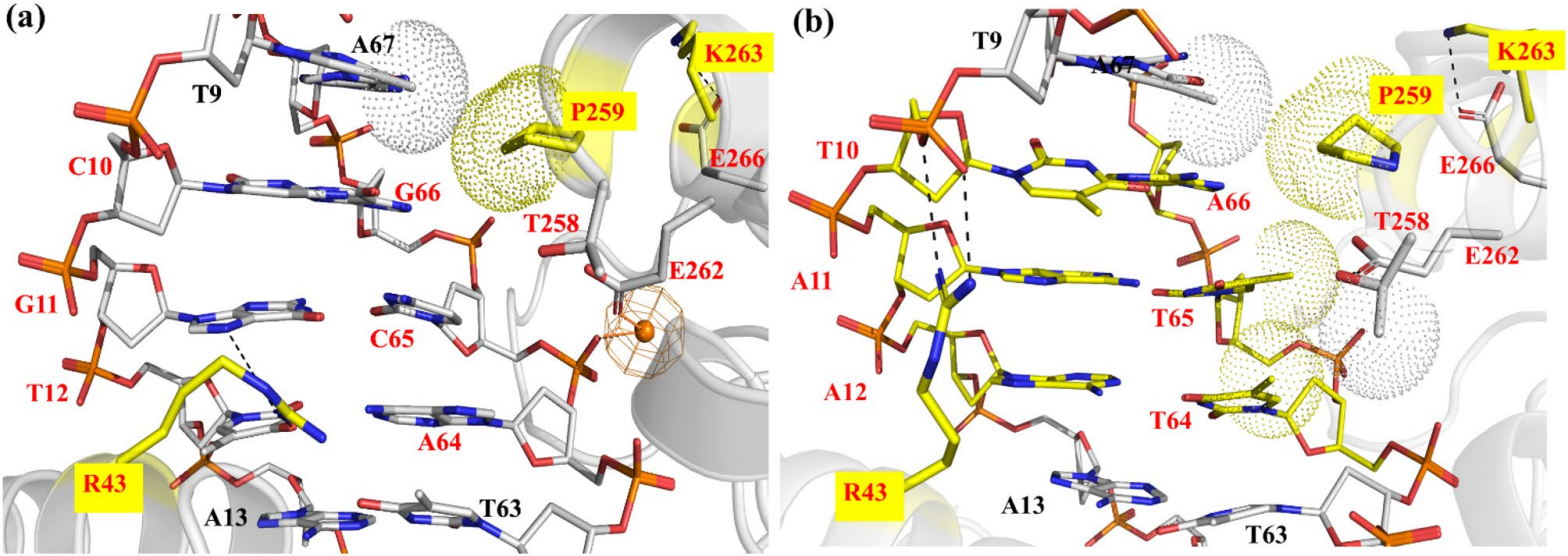
Detailed view of interactions observed in the (**a**) mCre_K_/*loxP* complex and (**b**) mCre_K_/*rox* complex at the PDI_BJ_ area based on MD simulations. The mutated amino acids with respect to wild type Cre are labeled with a yellow box. The hydrogen bonds are shown in black dashed lines and the van der Waals interactions in dotted spheres. A predicted water position using WaterMap (version 1.0, https://www.schrodinger.com/) is represented by an orange sphere and its interactions in solid orange lines. Pymol was used to generate the image (version 2.1, https://pymol.org/).

We next modeled the 3D structure of mCre_K_ (K43R, R259P and G263K) in complex with *loxP* and with *rox* in order to establish a structure–function rationale that could guide further engineering of mCre_K_ with improved activity on *rox*. The obtained mCre_K_/*loxP* and mCre_K_/*rox* complex structures were energy refined by MD simulations (see “Materials and methods” section for details). In the mCre_K_/*loxP* complex, R43 was observed to form bifurcated hydrogen bonding with N7 of G11 and O4 of T12, whereas in the mCre_K_/*rox* complex R43 interacted with the DNA backbone (Fig. 4a,b). Residue P259 formed compact van der Waals packing with the methyl groups of bases T65 and T64 on *rox*, along with residue T258. This compactness is missing in the complex with *loxP* due to the lack of those methyl groups in the altered bp C65 and A64 (Fig. 1a). Additionally, T258 was interacting with E262 in the mCre_K_/*rox* complex via hydrogen bonding. Residue K263 was observed to establish interactions with E266 in both mCre_K_/*loxP* and mCre_K_/*rox* complexes. However, a water-mediated contact was observed in the mCre_K_/*loxP* complex bridging E262 and the DNA backbone, while no such contact was detected in the mCre_K_/*rox* complex (Fig. 4). Altogether, the mCre_K_/*loxP* complex exhibited fewer interactions in the PDI_BJ_ area than mCre_K_/*rox*, providing a rationale for the altered selectivity of mCre_K_ on *loxP* and *rox*. The ion distributions in these complexes are presented in Fig. S2c and d. In the mCre_K_/*loxP* complex, a high K^+^ density is observed near the entrance of the major groove as mentioned above for the Cre/*loxP* complex, which could be associated to the presence of a GC-rich area, whereas in the mCre_K_/*rox* complex K^+^ density is observed only between the negatively charged groups of glutamic acid at position 266 and the DNA phosphates.

### Rational engineering of new Cre variants with enhanced activities on *rox*

The detailed analysis of the MD simulations suggested that amino acids on helix J other than those at positions 259 and 263 might play an important role in molecular recognition in the mCre_K_/*rox* complex. Therefore, we investigated in detail the possible implication in the recombination activity of residues in close proximity to specificity-determining ones in the PDI_BJ_ area.

Based on the hypothesis that neighboring residues to those determining specificity could potentially be used to tune activity, we focused on investigating in detail their recognition properties in order to select candidate positions for the introduction of new functionalities that could help in the engineering of improved recombination activity on *rox*. In the mCre_K_/*rox* complex, amino acid T258 was observed to form a hydrogen bond with the acceptor groups of E262, which also forced the latter to point towards the *rox* major groove (Fig. 4b). Thus, we hypothesized that by breaking the hydrogen bond between residues T258 and E262, we could increase the non-polar interactions at the PDI_BJ_ area by the reorientation of T258 towards the major groove and pushing E262 away from the groove. This way, E262 could then participate in interactions with K263 and/or with the DNA backbone and contribute towards stabilizing helix J. Hence, we decided to design several new variants of mCre_K_.

**Figure 5:**
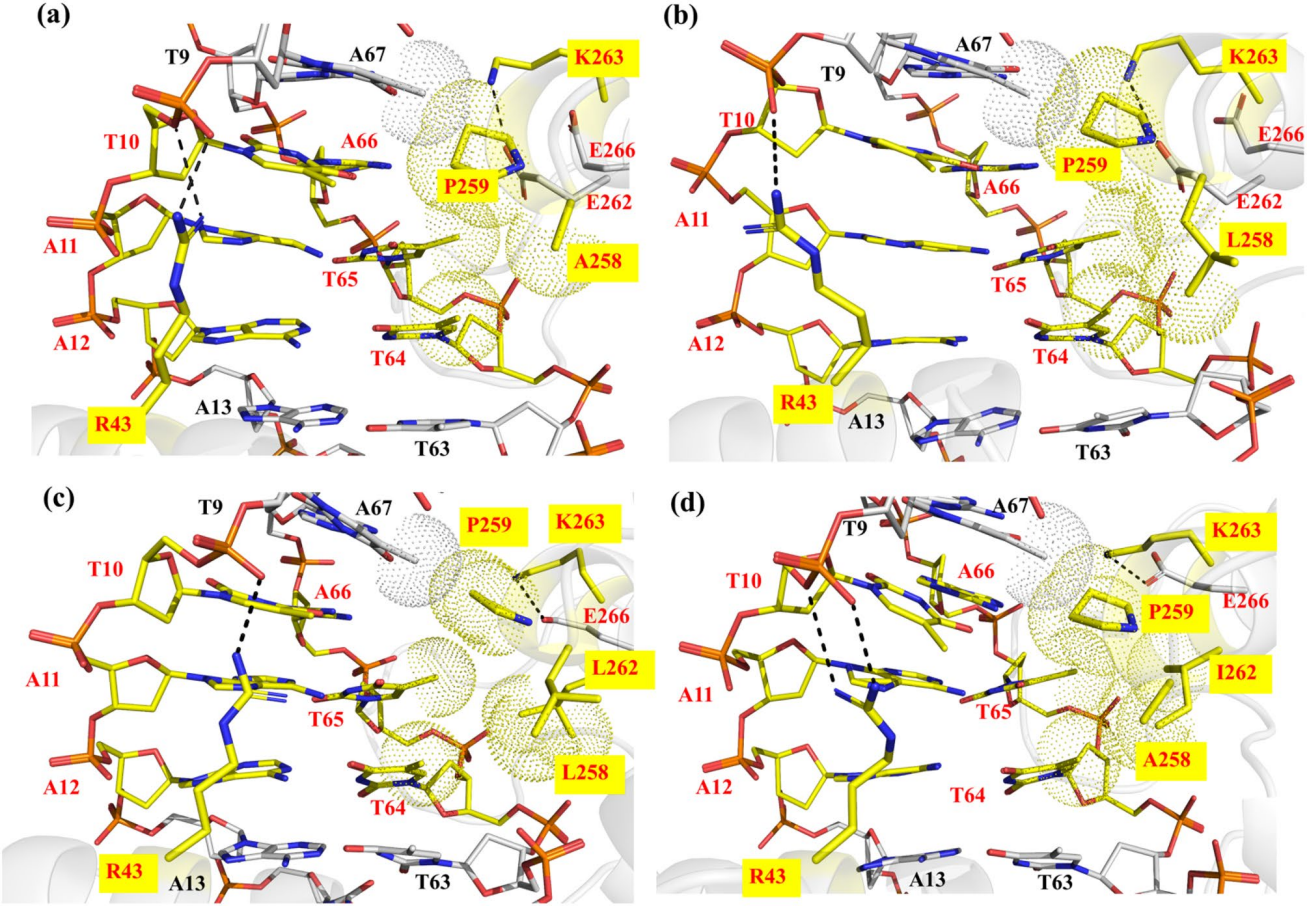
Detailed view of interactions observed at the PDI_BJ_ area in the MD simulations of the newly designed Cre variants with *rox*. (**a**) mCre1 (K43R, R259P, G263K, T258A) (**b**) mCre2 (K43R, R259P, G263K, T258L) (**c**) mCre3 (K43R, R259P, G263K, T258L, E262L) and (**d**) mCre4 (K43R, R259P, G263K, T258A, E262I). The mutated amino acids with respect to wild type Cre are labeled (yellow box). The hydrogen bonds are shown in black dashed lines and van der Waals interactions in dotted spheres. Pymol was used to generate the image (version 2.1, https://pymol.org/).

We designed a first new variant, mCre1, by introducing the mutation T258A in the mCre_K_ structure, which consists of a change to the Dre equivalent residue (mCre1; K43R, R259P, G263K, T258A) (see “Materials and methods” for details). The MD-based analysis of the mCre1/*rox* complex showed that the side chain of A258 promotes hydrophobic interactions with the methyl group of DNA base T65 (Fig. 5a). In this mCre1 variant, residues R43 and P259 were interacting with DNA bases T10 and A67, respectively, in a similar fashion as observed in the mCre_K_/*rox* complex (Figs. 4b, 5a). As hypothesized from our structure-based rationale, residue E262 was relocated pointing away from the groove and interacting with K263, and thus providing stability to the helix J. This displacement of E262 created a little void in the groove, which allowed the incorporation of a side chain bulkier than alanine at position 258. Therefore, we designed a second variant, mCre2, by introducing the mutation T258L (mCre2; K43R, R259P, G263K, T258L). The analysis of the results obtained in the MD simulation of mCre2/*rox* showed better packing in the groove by filling the void and involving T65 and T64 bases of *rox* while maintaining all the above-mentioned interactions (Fig. 5b). Next, while keeping the mutation T258L, and in order to further promote van der Waals and hydrophobic contacts, we designed another new mutant variant, mCre3, including the mutation E262L (mCre3; K43R, R259P, G263K, T258L, E262L). Simulation analysis of the mCre3/*rox* complex structure indicated that the mutations T258L and E262L had caused steric hindrance with the DNA bases and other protein residues, which enforced residues L258 and L262 to reorient and repack themselves with the adjacent hydrophobic residues L261, F265, I174 and A175 (Fig. 5c). Thereby, mCre3 resulted in a loose packing at the interface (Fig. 5c). To relieve possible steric repulsions, we designed a next variant, mCre4, in which position 262 was mutated to Isoleucine. Our molecular models indicated that the best counterpart for this change would be Alanine at position 258 (mCre4; K43R, R259P, G263K, T258A, E262I). Besides relieving the steric repulsions, we expected that these concurrent mutations in mCre4 would also provide some flexibility to helix J as noticed in the mCre1 variant (having T258A). The results obtained from the MD simulation analysis of the mCre4/*rox* complex showed the desired hydrophobic packing with the DNA bases A67, T65, and T64 (Fig. 5d). Overall, the mCre4/*rox* complex exhibited the highest number of interactions and the best packing complementarity. Water-mediated interactions were not observed in these newly engineered complexes. The ion distributions in these variants are shown in Fig. S2e–h. These variants also displayed high K^+^ densities between negatively charged E266 and DNA phosphates as observed in all the above complexes to minimize charge repulsion.

Interestingly, in our simulations we observed that when E262 is mutated to hydrophobic residues (i.e. Leucine and Isoleucine in mCre3 and mCre4, respectively), its role in binding to K263 is taken over by residue E266, thereby providing stability to helix J. This interaction was also observed in the mCre_K_/*rox* complex. The observation of mutating-neighboring residues taking over the role of indispensable residues has also been previously reported in evolved SSR systems^37^. Our MD-based analyses strongly emphasize on the relevance of having non-polar residues at certain neighboring positions in the PDI_BJ_ area, which could possibly affect activity.

In our rationale, when we hypothesized the breaking of the hydrogen bond between T258 and E262 in the mCre_K_/*rox* complex, we proposed the simplest mutation of Threonine to Alanine at position 258 (as in mCre1 variant). As mentioned above, we then thoroughly investigated the impact of diverse amino acids on molecular recognition by introducing the mutation at position 258 alone and in combination with position 262, which led us to the use of hydrophobic residues i.e. Leucine and Isoleucine (as in mCre2, mCre3, and mCre4 variants). The MD analyses showed that the inclusion of these hydrophobic residues enhances the complementary packing at the DNA interface, mostly with the methyl groups of bases T65 and T66 in *rox*. The exception to this observation was the mCre3 variant. Coincidently, the sequence alignment of Cre, Dre and other related naturally occurring SSRs also revealed the presence of Alanine at position 258 in Dre and Panto, whereas the other SSRs present Threonine, Proline or Glutamic acid at this position (Fig. S4). However, none of the naturally occurring SSRs harbors bulky hydrophobic/non-polar residues at position 258 and/or 262. In fact, position 262 is occupied in most cases by charged/polar residues. This observation was particularly interesting for Dre and Panto, as they have Thymine as a base at positions 65 and 66 in their respective target sites. Our findings from the MD analysis of the new Cre variants underscore the presence of bulky hydrophobic groups at positions 258 and 262 with respect to *rox*. We further decided to investigate these findings energetically.

### MD-based energetic analyses

In order to gain a deeper understanding on the recognition properties of the selected mutations and their potential effect on recombination activities, we estimated binding energies of all the respective protein-DNA complexes (mCre_K_/*loxP*, mCre_K_/*rox*, mCre1/*rox*, mCre2/*rox*, mCre3/*rox* and mCre4/*rox*). For this purpose, we performed a comparative energetic analysis of the mutant variants with respect to the wild type complexes (Cre/*loxP*, Dre/*rox*) utilizing MM-GB/PBSA^38^. The predicted MM-GB/PBSA binding energy of the Cre/*loxP* complex was higher than for the Dre/*rox* complex (− 662.61/− 781.01 versus − 465.78/− 577.23 kcal/mol), which is due to its greater number of contacts (Table 1 and Fig. S1). The calculated MM-GB/PBSA energies also reflected stronger binding of mCre_K_ to *rox* than to *loxP* (− 657.54/− 785.05 and − 632.64/− 772.92 kcal/mol, respectively), as observed in the MD-based molecular analyses of the corresponding complexes.

**Table 1:** Calculated MM-GB/PBSA binding energies and corresponding standard deviations (kcal/mol) for the studied SSRs complexes.

Complexes	ΔE_vdW_	ΔE_ele_	ΔG_GB_	ΔG_PB_	ΔG_SA(GB)_	ΔG_SA(PB)_	ΔG_GBSA_	ΔG_PBSA_
Cre/loxP	− 533.3 ± 16.6	− 28,163.6 ± 274.2	28,110.2 ± 266.9	27,973.5 ± 266.1	− 75.8 ± 1.4	− 57.5 ± 0.6	− 662.6 ± 31.4	− 781 ± 29.2
Dre/rox	− 461.3 ± 15	− 21,026.5 ± 295	21,087.2 ± 282.1	20,962.0 ± 281.7	− 65.1 ± 1.6	− 51.4 ± 0.9	− 465.7 ± 33.8	− 577.2 ± 28.3
mCre_K_/loxP	− 494.9 ± 14.3	− 27,939.3 ± 337.8	27,873.7 ± 336.2	27,716.1 ± 331.1	− 72.1 ± 1.5	− 54.7 ± 0.9	− 632.6 ± 26.6	− 772.9 ± 24.4
mCre_K_/rox	− 519.3 ± 16.2	− 27,065.7 ± 286.6	27,003.1 ± 283.5	26,886.9 ± 288.9	− 75.6 ± 1.7	− 56.9 ± 0.8	− 657.5 ± 27.4	− 785.0 ± 26.1
mCre1/rox	− 510.0 ± 25.5	− 27,428.3 ± 330.7	27,388.7 ± 331	27,212.2 ± 334.8	− 72.1 ± 2.3	− 55.4 ± 1.3	− 621.7 ± 24.8	− 781.5 ± 23.8
mCre2/rox	− 515.7 ± 14.7	− 27,388.9 ± 290.4	27,358.3 ± 283	27,189.1 ± 288.1	− 72.8 ± 1.3	− 56.3 ± 0.8	− 619.1 ± 29.8	− 771.8 ± 33.4
mCre3/rox	− 530.3 ± 16.5	− 28,623.0 ± 235.1	28,565.3 ± 223.5	28,392.1 ± 227.1	− 74.9 ± 1.5	− 56.6 ± 0.7	− 663.1 ± 34.8	− 818.0 ± 31.6
mCre4/rox	− 542.3 ± 12.9	− 28,998.7 ± 289.4	28,939.4 ± 279.5	28,799.2 ± 281.9	− 76.1 ± 1.2	− 57.1 ± 0.6	− 677.8 ± 30.3	− 798.8 ± 26.1

*ΔE*_*vdW*_ van der Waals energy, *ΔE*_*ele*_ columbic energy, *ΔG*_*GB*_ Generalized-Born polar solvation energy, *ΔG*_*PB*_ Poisson–Boltzmann polar solvation energy, *ΔG*_*SA(GB/PB)*_ non-polar solvation energy from MM-GBSA/MM-PBSA.

**Figure 6:**
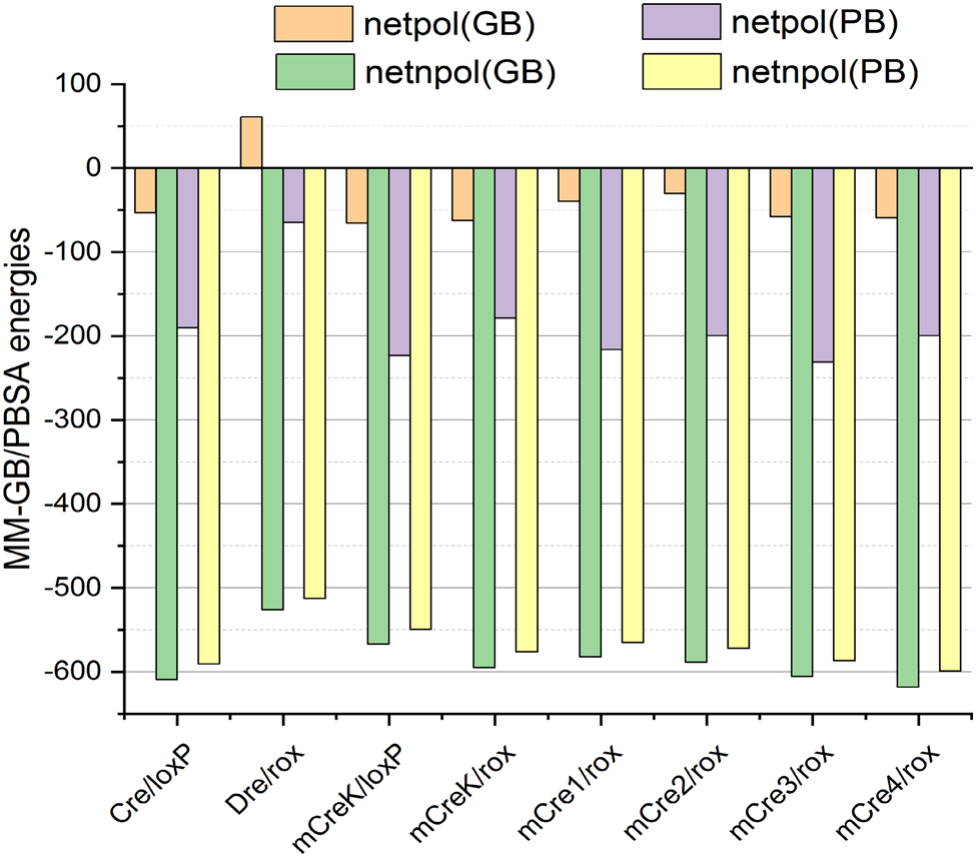
Net free energy component analyses of the studied complexes; net_pol(GB/PB)_: net polar contributions from MM-GBSA/MM-PBSA (net_pol(GB/PB)_ = ΔE_ele_ + ΔG_GB/PB_), net_npol(GB/PB)_: net non-polar contributions from MM-GBSA/MM-PBSA (net_npol(GB/PB)_ = ΔE_vdW_ + ΔG_SA(GB/PB)_).

The interaction energies obtained for the newly engineered Cre variants indicated that all the introduced mutations had a favorable effect on the molecular recognition of *rox* (mCre1: − 621.75/− 781.58, mCre2: − 619.15/− 771.85, mCre3: − 663.06/818.02 and mCre4: − 677.83/− 798.86 kcal/mol) compared to the native Dre/*rox* complex (Table 1). The binding energies of the new variants were not statistically very different from mCre_K_/*rox*, and the strongest contributions in all the cases were obtained from the net non-polar (net_npol_) components (Table 1 and Fig. 6). We decided to perform the experimental validation of all the four variants against *rox*.

### Experimental validation of rationally engineered new Cre variants

**Figure 7:**
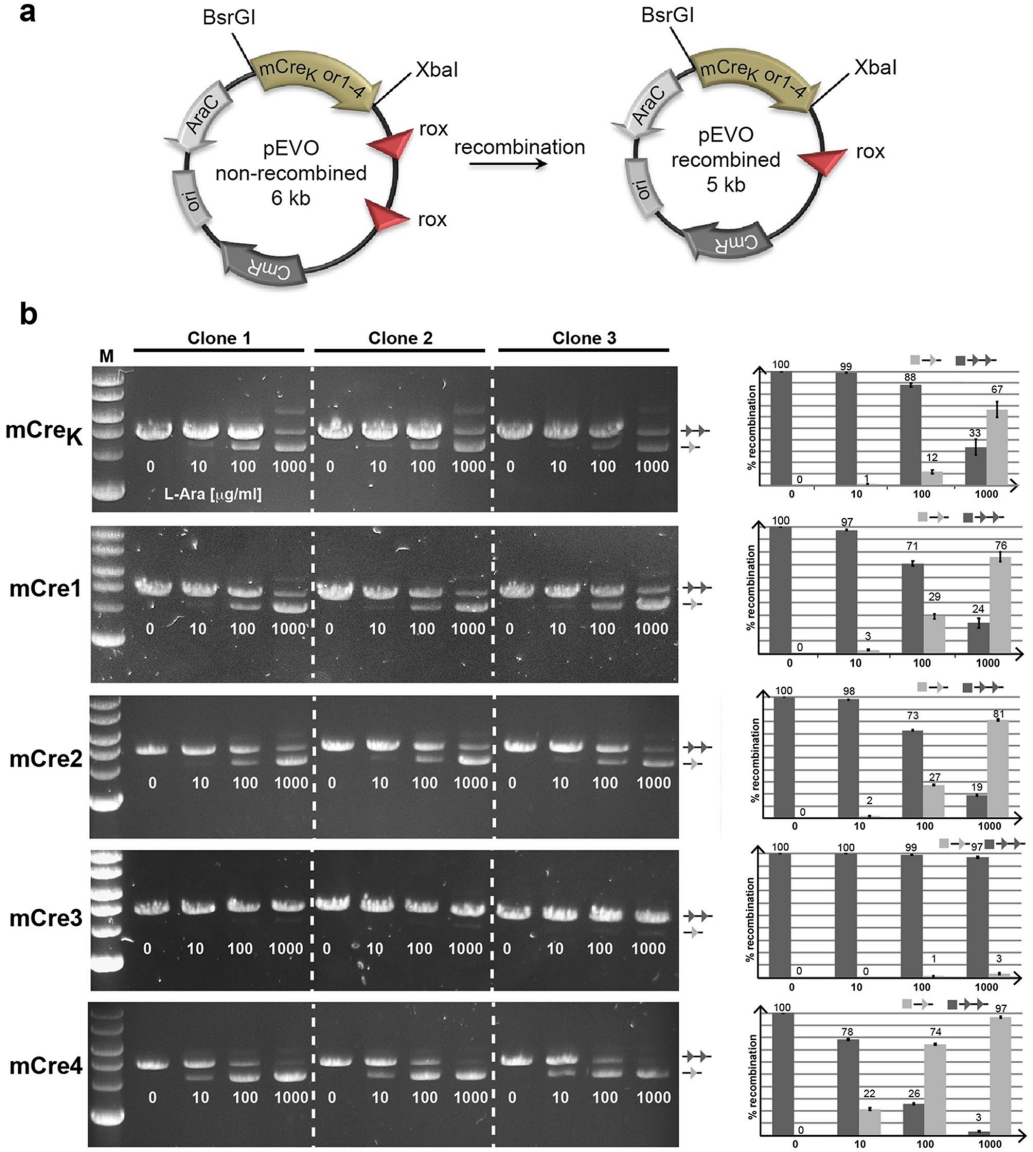
Rationally designed Cre mutants show increased recombination activity in *E.coli*. (**a**) Schematic drawing of the plasmid assay. Important regions in the plasmids are indicated. Note the reduced size of the plasmid after recombination. The restrictions sites (BsrGI and XbaI) used for cloning indicated Cre-recombinase variants are depicted. The *rox* target sites are shown as red triangles. CmR, chloramphenicol resistance gene; ori, origin of replication; AraC, arabinose operon regulatory gene. (**b**) Agarose gels of three independently picked clones showing BsrGI and XbaI digested plasmids carrying indicated recombinases. The amount of arabinose added to the growth medium is presented below each band in μg/ml l+-arabinose. The line with two triangles indicates no recombination, whereas the line with one triangle marks the recombined band. Quantifications of the ratios of band intensities (in percent of recombination) are shown to the right for each mutant. The amount of arabinose added to the growth medium is shown on the X-axis. Error bars depict standard deviation from the three independent experiments.

To test the newly designed Cre variants (mCre1, mCre2, mCre3 and mCre4) experimentally, we introduced the corresponding mutations into the mCre_K_ coding sequence. The sequences were confirmed by sequencing, and the recombinase mutants were cloned into the pEVOrox vector that allows regulated expression of the recombinase enzymes^12^. The vector also harbored two *rox* sites in direct orientation as an excision substrate (Fig. 7a), making it possible to investigate recombinase activity by the growth of the plasmids in bacteria at different l-arabinose concentrations followed by plasmid extraction, digestion and gel electrophoresis. Growing pEVOrox-mCre_K_ at different l-arabinose concentrations confirmed that the three mutations introduced into Cre (K43R, R259P, G263K) conveyed recombination activity on the *rox* target sites, although partial recombination was only visible at high l-arabinose concentrations (Fig. 7b). Changing the threonine at position 258 to alanine (mCre1) or leucine (mCre2) slightly increased the recombination activity, but when the position 262 was changed to leucine in combination to L258 (mCre3), the recombination activity was completely lost. The loss in activity could be associated to the steric repulsions and poor interfacial interactions as observed through in silico analyses. Nevertheless, a remarkable increase in recombination activity was observed when position 262 was changed to Isoleucine in conjunction with A258 (mCre4). Indeed, full recombination of the plasmid was observed at the highest l-arabinose concentration for this variant (Fig. 7b), with increased recombination activity of up to 20-fold observed on the rox site when compared to mCre_K_ (Supplementary Fig. S5). These data experimentally validate our structure-based rationale and MD simulation results, confirming that residues next to specificity-determining amino acids influence the activity of Cre-type site-specific recombinases.

## Conclusions

To better understand how Cre-type site-specific DNA recombinases may achieve better precision in terms of recombination activity, we investigated in detail the protein-DNA recognition properties of such recombinase systems in a comparative fashion by applying molecular modeling and dynamics simulations. Molecular dynamics simulations and binding energy calculations were used to examine by molecular and energetic means the mechanisms involved in DNA target recognition in the naturally occurring recombinase systems Cre/*loxP* and Dre/*rox* and the engineered variant mCre_K_. Although being able to recombine *rox*, mCre_K_ exhibited low recombination activity, which made us consider a detailed analysis of the recognition properties of these recombinase systems in the region in which the specificity-determining residues are located (PDI_BJ_ area) and to scrutinize at atomic level any aspect that may affect activity. The analysis of our theoretical molecular models and MD simulations pointed to neighbor amino acids of specificity-determining residues as relevant contributors to DNA target recognition and, therefore, as promising candidate positions to be exploited for the rational design of improved recombination activities. In particular, our MD-based analyses strongly emphasized on the relevance of having non-polar substitutions at positions 258 and 262 in the PDI_BJ_ area, a feature not observed in naturally occurring SSRs. We established a rationale to account for the structure–function relationships, which we used to design new Cre variants predicted to have improved recombination activity on *rox*. The experimental validation of the newly designed Cre variants confirmed our predictions and supported the hypothesis that changes in the nature of amino acids spatially close to the specificity-determining residues could lead to enhance activities of engineered SSRs. This work demonstrates that computer-aided molecular modeling and simulation are valuable tools to build up innovative rational strategies for the efficient engineering of SSR systems with desired properties for applied site-specific recombination.

The results obtained should help for the future generation of designer-recombinases. Several amino acids in particular regions in Cre-like designer recombinases have been classified as implicated in the specificity of the enzymes^28,37,39,40^. DNA shuffling is currently used to combine beneficial mutations, thereby accelerating the directed evolution process. However, because DNA shuffling relies on the homology of DNA fragments, this method is not very efficient in combining residues that are close in sequence. Our results argue that amino acids that flank specificity-determining residues have an important role in obtaining SSRs with the highest activity. We, therefore, propose that targeted mutagenesis of nearest-neighbor amino acids of specificity-determining residues should be performed to optimize the activity of engineered SSRs.

## Materials and methods

### Molecular modeling and MD simulations

The crystal structure of the Cre/*loxP* complex was obtained from RCSB Protein Data Bank^32^ (PDB 1Q3U^19^, resolution 2.9 Å). This structure consists of four molecules of Cre and two of the *loxP* target site. For simplicity, in our modeling and dynamics simulations we have used half of the system (i.e. one *loxP* and two Cre molecules; cleaving and non-cleaving). This structure together with other available structural homologs at the Protein Data Bank (PDBs: 5U91, 1KBU, 3CRX) were used as a template to generate a 3D model of the Dre protein. For this, we used the comparative/homology modeling tool of Discovery Studio (DS version 3.5, https://www.3dsbiovia.com/)^26^ and MODELLER^33,34^ as implemented in DS. The SWISS-MODEL (version 1.0, https://swissmodel.expasy.org/)^35^ and PHYRE2 (version 2.0, https://www.sbg.bio.ic.ac.uk/)^36^ webservers with default values were also used in order to generate further 3D models of Dre. Likewise, the 3D model of the DNA target *rox* was generated using the *loxP* structure as a template with the modeling tool of DS. The Dre/*rox* complex was obtained by manual docking based on the superposition of the modeled Dre and *rox* molecules with the Cre/*loxP* structure while keeping the catalytic tyrosine and phosphate in close proximity. The 3D structures of all Cre mutant variants (mCre_K_, mCre1, mCre2, mCre3, and mCre4) were also obtained using the homology modeling tool of DS, and PDB 1Q3U was used as template. Similar manual docking procedures by superposition with the Cre/*loxP* structure were used to generate the 3D models of all Cre mutant variants in complex with the DNA targets (mCre_K_/*loxP*, mCre_K_/*rox*, mCre1/*rox*, mCre2/*rox*, mCre3/*rox,* and mCre4/*rox*). Hydrogen atoms were added to the complexes (including the wild type crystallographic structure of Cre/*loxP*) using the leap module of AMBER14 (https://ambermd.org/)^41^, and force-field parameters were assigned to the protein and DNA using ff14SB^42^ and parmbsc1^43^ force-fields, respectively. Energy refinement of all complexes was carried out by using molecular dynamics (MD) simulations adopting ABC (Ascona B-DNA Consortium) protocols^44,45^.

MD simulations were performed on all the complexes with periodic boundary conditions in a truncated octahedral cell using the AMBER14 software suite^41^. The protein/DNA complexes were solvated with SPC/E^46^ water molecules, and charge neutrality was maintained by adding a sufficient number of potassium ions to the system. Simulations were conducted with 0.15 M KCl concentration using parameters from Dang^47^. Counterions were randomly placed initially in a cell, but no less than 5 Å away from DNA and 3.5 Å from one another. Electrostatics were handled using the Particle Mesh Ewald method^48^ with a cutoff of 10 Å. Lennard–Jones interactions were truncated at 9 Å. Initial energy minimization of the solvent (2,500 steepest descent and 2,500 conjugate gradient) was performed with harmonic restraints of 25 kcal mol^−1^ Å^−2^ on the solute, and then the minimization of solute–solvent was performed. Followed by minimization, equilibration was performed with slow heating of the solvent to 300 K at constant volume for a period of 200 ps, while restraining the solute atoms by 25 kcal mol^−1^ Å^−2^. These positional restraints were gradually removed from 5 to 1 kcal mol^−1^ Å^−2^ during the series of minimizations and equilibrations over a period of 1 ns. Finally, the production simulations were carried out initially for 100 ns using an NPT ensemble and the Berendsen algorithm^49^, which were later extended to 200 ns. All bonds involving hydrogen were constrained using SHAKE^50^.

### Structural and energetic analyses of Protein–DNA molecular recognition

All the studied protein–DNA complexes remained structurally stable during the entire MD simulations as illustrated by the RMSD values obtained through the simulation time (Fig. S6). The structure-based analysis of the direct and indirect (water-mediated) hydrogen bonding established between the protein and DNA molecules in the studied complexes was done using the cpptraj^51^ module of AMBER14 and the WaterMap^27^ tool of Schrodinger (version 1.0, WaterMap, Schrödinger, 2019; https://www.schrodinger.com/). WaterMap is based on the inhomogeneous solvation theory, which analyses the results based on a short (2 ns) MD simulation. During MD, the complex is held rigid and water molecules are allowed to move. The clustering analysis is then performed on the population of water molecules to predict the location, and the free energy of each (favorable and unfavorable) water site is calculated. As the predictions are done on the population density of water molecules, the residence times of individual waters are not reported. Nucplot^52^ was also used for hydrogen bonding and van der Waals analyses. VMD^53^ was used for trajectory analysis. Pymol was used for the generation of figures (version 2.1, https://pymol.org/). The average K^+^ distribution analyses were performed using the Grid command of CPPTRAJ, AMBER14. The energetic analysis consisted of binding enthalpies calculated from the last 50 ns of the initial MD simulation using the MM-GB/PBSA^38^ module of AMBER (200 snapshots were used for calculations). The results represented in Table 1 and Fig. 6 belong to the initial 100 ns MD trajectory. The free energy analyses on the extended trajectory (100 ns to 200 ns) showed similar results (Table S3). The structural analyses performed for the extended simulations confirmed the interaction details obtained with the frames extracted from the initial MD trajectory and shown in Figs. 2, 3, 4 and 5.

### Recombination assays in *E. coli*

For expression in *E. coli*, mCre_K_ and variants thereof were cloned into the pEVOrox vector^12^ utilizing the unique BsrGI and XbaI (NEB, Ipswich, MA, USA) restriction sites. To introduce mutations into the mCre_K_ coding sequence, site-directed mutagenesis was performed using the Q5 Site-Directed Mutagenesis Kit (NEB, Ipswich, MA, USA) following the manufacturer’s instructions. The expression of the recombinases from the pBAD promoter was induced with l-(+)-arabinose (Sigma-Aldrich Chemie GmbH). Single colonies of XL1-blue *E.coli* (recA1 endA1 gyrA96 thi-1 hsdR17 supE44 relA1 lac [F′ proABlacIqZ∆M15 Tn10 (Tetr)]; Agilent, Santa Clara, CA, USA) containing pEVOrox plasmid with the recombinase and recombination target sites were cultured overnight in 5 ml Luria broth (LB) medium with 30 μg/ml Cm and 0, 10, 100 or 1,000 μg/ml l-(+)-arabinose at 37 °C and 200 rpm before plasmid extraction, digestion, and gel electrophoresis. Gels were run to best separate the non-recombined forms and the recombined forms of the plasmids. The ca. 1 kb bands of the recombinase are therefore not visible in the gels in Fig. 7. Experiments were repeated with two additional clones and bands corresponding in size to the non-recombined and the recombined vector were quantified with Fiji–win64 software (ImageJ), respectively. The sum of non-recombined and recombined area was regarded as 100% and the fraction of non-recombined and recombined areas were calculated in percent. The standard deviations (STDDEV) of the 3 clones carrying the same mutations at identical l-(+)-arabinose induction levels were calculated.

## Supplementary information

Supplementary Information.
