# Supplementary material for: Nearest-neighbor amino acids of specificity-determining residues influence the activity of engineered Cre-type recombinases
Source: Sci Rep. 2020 Aug 19;10:13985. doi: 10.1038/s41598-020-70867-5 (PMC7438526; doi:10.1038/s41598-020-70867-5)
Supplement: Supplementary file 1 — Supplementary Information. [file 41598_2020_70867_MOESM1_ESM.pdf]

Nearest-neighbor amino acids of specificity-determining residues influence the activity of engineered Cre-type recombinases

Anjali Soni[1], Martina Augsburg[2], Frank Buchholz[2,] and M. Teresa Pisabarro[1,*]

[1]Structural Bioinformatics, BIOTEC, TU Dresden, Tatzberg 47-51, 01307 Dresden, Germany

[2]University Carl Gustav Carus and Medical Faculty, UCC, Medical Systems Biology, TU Dresden, Fetscherstrasse 74, Dresden, Germany

* Corresponding author. Correspondence to maria_teresa.pisabarro@tu-dresden.de

Table of contents

Fig. S1	Sequence alignment of Cre and Dre recombinases is shown along with their secondary structure. Dissimilar amino acids are boxed in red. Protein-DNA interactions in Cre/loxP (red) and Dre/rox complexes (blue) are analyzed from MD simulations. Diamonds represent the backbone (phosphate and sugar) interactions and stars represent the interactions to the bases.	3
Table S1	Hydrogen bonds observed in Cre/loxP complex shown as percentage occurrence for one monomer during 100ns of simulations. The comparisons are made with the interactions in PDB 1Q3U. Black represent the interactions common to crystal structure and the MD simulation, green represent the interactions seen only in the crystal structure and red represent the interactions seen only in MD simulations.	4
Fig. S2	Average K+ distribution observed in all the studied complexes at the investigated PDI_{BJ} area. The three nucleotides that differ between *loxP* and *rox* are colored gray and labeled. The helix B of protein is represented in magenta and helix J in green. The K+ densities are represented as isosurface in blue.	5
Fig. S3	Structural superposition of the crystal structure of the Cre/loxP complex (PDB 1Q3U) with the Dre/rox models obtained by different computational means (MODELLER/Discovery Studio (DS), Swiss-Model and Phyre2). The two monomer units of the recombinases are represented in cartoon and the DNA is shown in ladder view.	6
Table S2	Hydrogen bonds observed in Dre/rox complex shown as percentage occurrence for one monomer during 100ns of simulations. Due to the unavailability of the crystal structure, interactions are marked red as occurs only in MD simulations.	7
Fig. S4	Multiple sequence alignment of nearest-neighbor amino acids on helix J of naturally occurring Cre-like SSRs.	8
Fig. S5	Recombination activity of indicated mutants in comparison to $mCre_K$ on loxP and rox. Fold changes were calculated from band intensities from gels shown in Figure 7 at 10 μg/ml L-arabinose. Error bars show the standard deviation from three independent experiments.	8
Fig. S6	Root mean square deviation (RMSD) with respect to time for the studied complexes.	9
Table S3	Calculated MM-GB/PBSA binding energies (kcal/mol) and corresponding standard deviations for the studied SSRs complexes (for a period of 100 to 200 ns).	9

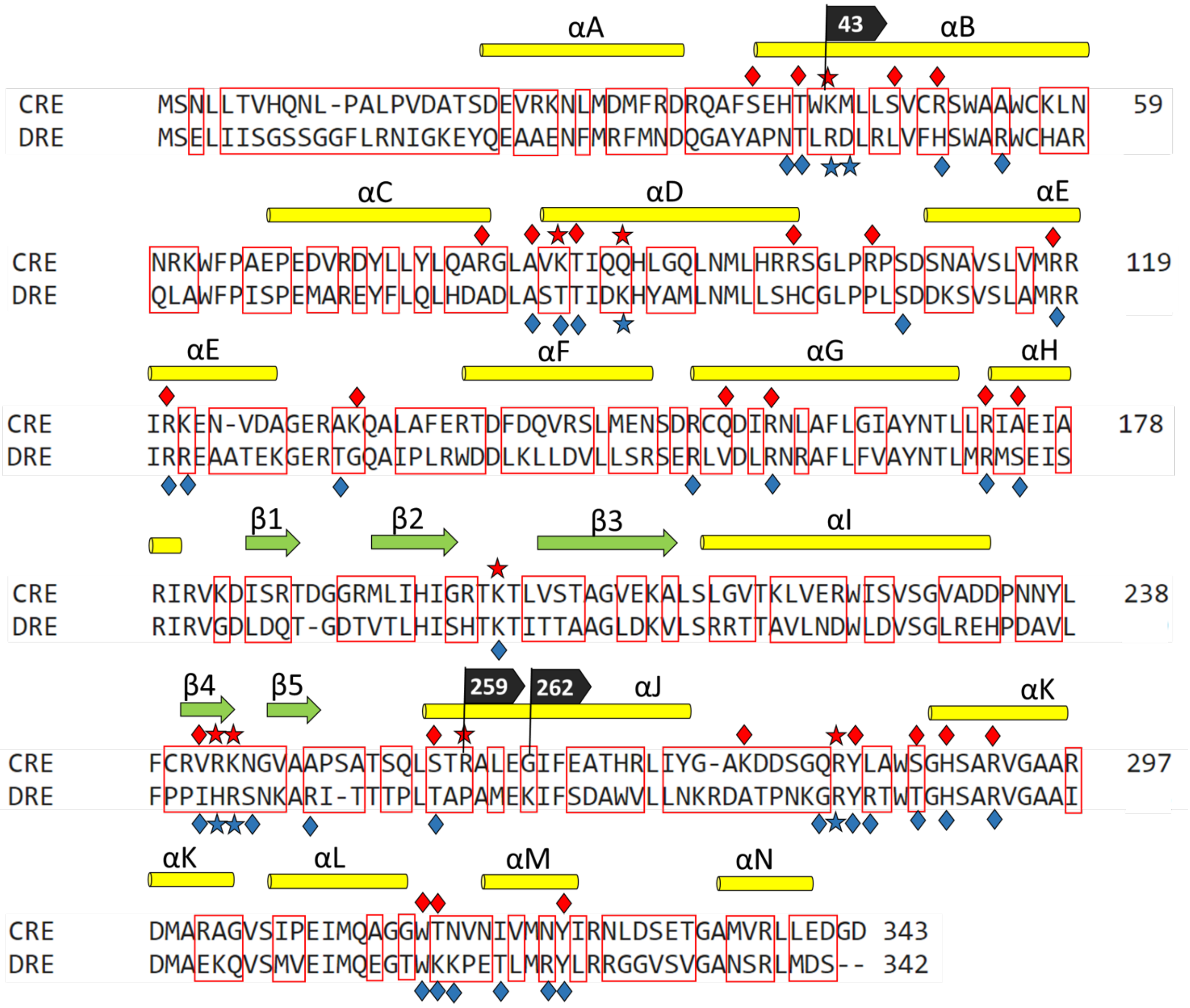

Fig. S1. Sequence alignment of Cre and Dre recombinases is shown along with their secondary structure. Dissimilar amino acids are boxed in red. Protein-DNA interactions in Cre/loxP (red) and Dre/rox complexes (blue) are analyzed from MD simulations. Diamonds represent the backbone (phosphate and sugar) interactions and stars represent the interactions to the bases.

Table S1. Hydrogen bonds observed in Cre/loxP complex shown as percentage occurrence for one monomer during 100ns of simulations. The comparisons are made with the interactions in PDB 1Q3U. Black represent the interactions common to crystal structure and the MD simulation, green represent the interactions seen only in the crystal structure and red represent the interactions seen only in MD simulation.

Protein	Base	% Occurrence	Protein	Base	% Occurrence
LYS_43@NZ	DT_63@O4	-	LYS_244@NZ	DT_4@O2	84
LYS_43@NZ	DG_11@N7	73	LYS_244@NZ	DT_73@O2	69
LYS_86@NZ	DA_16@N7	51	ARG_259@NH2	DG_66@N7	55
GLN_90@NE2	DT_61@O4	80	ARG_259@NE	DG_66@O6	40
GLN_90@NE2	DT_14@O4	63	ARG_282@NH2	DA_13@N3	70
LYS_201@NZ	DA_16@N3	42	ARG_282@NH1	DT_14@O2	31
LYS_201@NZ	DT_61@O2	-	ARG_282@NH1	DT_63@O2	15
ARG_243@NH1	DT_71@O2	28	ASN_319@ND2	DA_55@N7	26

Protein	Backbone (P)	% Occurrence	Protein	Backbone (P)	% Occurrence
SER_38@N	DT_61@OP2	87	ARG_173@NH1	DT_63@OP1	33
SER_38@OG	DT_61@OP2	25	ARG_173@NH2	DT_17@OP1	82
SER_38@OG	DA_62@OP2	57	ALA_175@N	DT_63@OP1	46
THR_41@OG1	DT_61@OP2	98	ARG_199@NH2	DA_62@OP2	24
SER_47@OG	DT_12@OP2	77	ARG_199@NE	DA_62@OP1	20
ARG_50@NH2	DT_12@OP1	11	THR_200@OG1	DG_18@OP1	32
ARG_50@NH2	DT_12@OP2	13	VAL_242@N	DC_7@OP1	65
ARG_81@NH1	DA_13@OP1	20	ARG_243@NE	DT_73@OP1	13
ARG_81@NH1	DA_13@OP2	36	ARG_243@NH2	DT_73@OP1	32
ARG_81@NH2	DA_13@OP1	56	SER_257@N	DT_8@OP2	78
ARG_81@NH2	DA_13@OP2	17	SER_257@OG	DT_8@OP2	98
ALA_84@N	DT_14@OP2	80	ARG_259@NH1	DC_65@OP2	19
THR_87@OG1	DT_14@OP2	97	LYS_276@NZ	DG_66@OP2	41
ARG_100@NH1	DT_60@OP2	33	LYS_276@NZ	DG_128@OP1	-
ARG_100@NH2	DT_60@OP2	37	TYR_283@OH	DA_16@OP1	31
ARG_100@NH1	DA_59@OP1	20	TYR_283@N	DC_65@OP1	11
ARG_106@NE	DA_59@OP2	32	SER_287@HG	DA_64@OP1	99
ARG_106@NH1	DC_58@OP1	40	HIS_289@N	DA_64@OP1	47
ARG_106@NH2	DA_59@OP2	80	ARG_292@NE	DT_17@OP1	42
ARG_106@NH2	DC_58@OP2	14	ARG_292@NH1	DT_17@OP1	16
SER_108@OG	DC_58@OP2	18	ARG_292@NH2	DT_17@OP1	42
ARG_118@NH1	DC_23@OP1	88	ARG_292@NH2	DT_17@OP2	42
ARG_118@NH2	DC_23@OP1	31	TRP_315@NE1	DT_17@OP2	75
ARG_121@NH1	DA_57@OP1	77	THR_316@OG1	DG_18@OP1	40
LYS_132@N	DA_15@OP1	48	THR_316@N	DG_18@OP1	74
GLN_156@NE2	DC_7@OP2	50	ASN_317@N	DG_18@OP2	66
ARG_159@NH1	DC_7@OP1	96	TYR_324@OH	DT_17@OP2	68
ARG_159@NH2	DC_7@OP2	94	TYR_324@OH	DA_16@OP1	-
ARG_173@NE	DT_17@OP1	40	-		

Protein	Backbone (S)	% Occurrence	Protein	Backbone (S)	% Occurrence
THR_87@OG1	DT_14@O5'	-	ARG_282@NH1	DA_15@O4'	23
ARG_106@NH2	DC_58@O5'	19	THR_316@OG1	DG_18@O5'	10

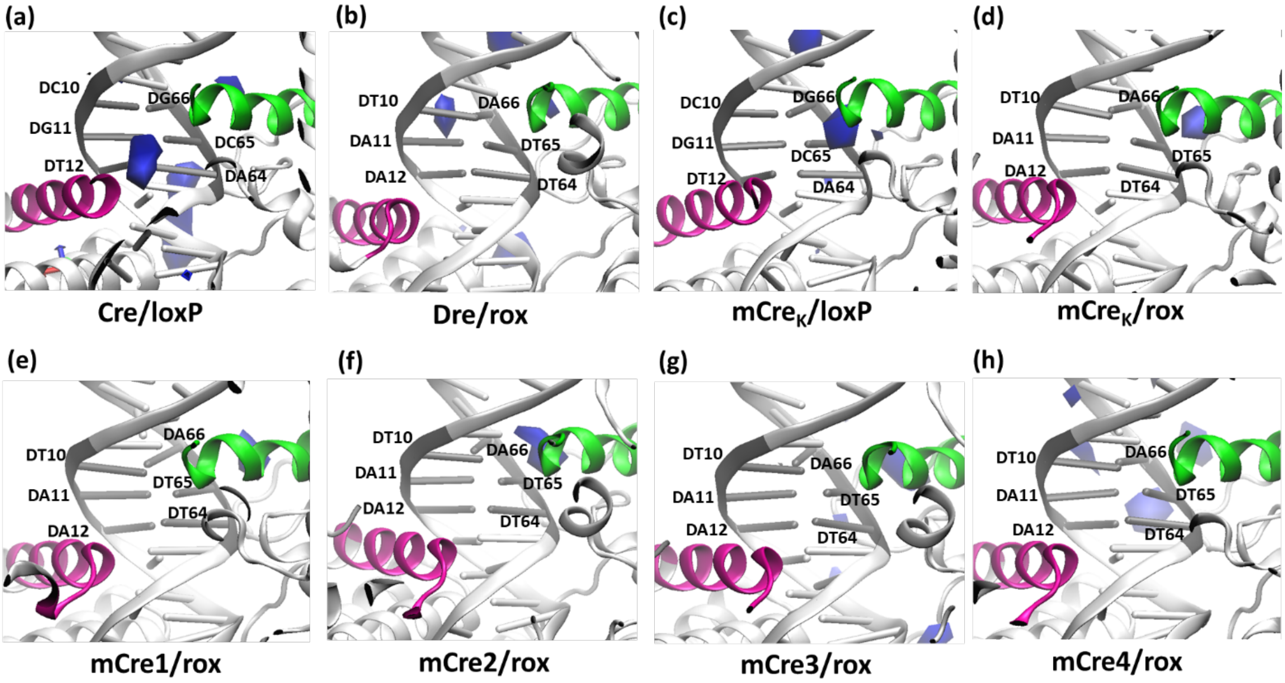

Fig. S2. Average K+ distribution observed in all the studied complexes at the investigated PDI_{BJ} area (predicted using Grid module of Amber). The three nucleotides that differ between *loxP* and *rox* are shown as gray sticks and labeled. The protein helices B and J are shown in magenta and green ribbons, respectively. The K+ densities are as indicated with a blue isosurface. VMD was used to generate the image.

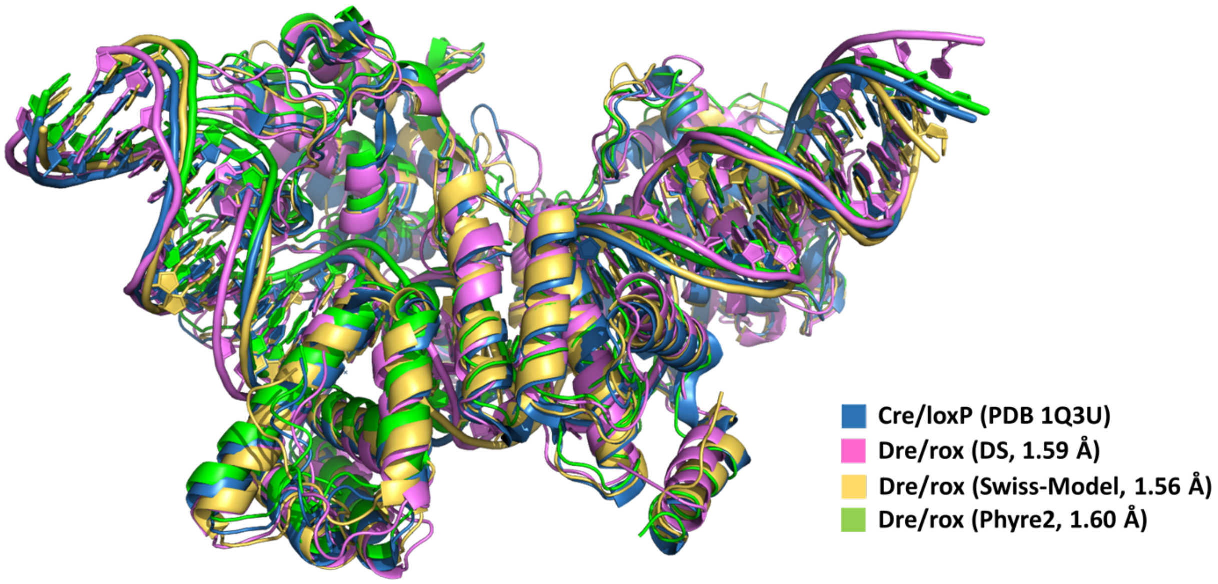

Fig. S3. Structural superposition of the crystal structure of the Cre/loxP complex (PDB 1Q3U) with the Dre/rox models obtained by different computational means (MODELLER/Discovery Studio (DS), Swiss-Model and Phyre2). The two monomer units of the recombinases are represented in cartoon and the DNA is shown in ladder view. Pymol was used to generate the image.

Table S2. Hydrogen bonds observed in Dre/rox complex shown as percentage occurrence for one monomer during 100ns of simulations. Due to unavailability of the crystal structure, interaction are marked red as occurs only in MD simulation.

Protein	Base	% Occurrence	Protein	Base	% Occurrence
ARG_43@NH1	DT_63@O4	70	LYS_90@NZ	DT_61@O4	35
ARG_43@NH2	DT_63@O4	14	HIS_243@NE2	DA_72@N3	22
ASP_44@OD1	DA_62@N6	47	ARG_244@NH2	DT_4@O2	19
LYS_90@NZ	DT_14@O4	48	ARG_282@NH2	DT_14@O2	12

Protein	Backbone (P)	% Occurrence	Protein	Backbone (P)	% Occurrence
ASN_40@ND2	DA_62@OP2	16	LYS_201@NZ	DA_62@OP1	19
THR_41@OG1	DT_61@OP2	67	ILE_242@N	DC_7@OP1	49
HIS_50@NE2	DA_12@OP2	13	SER_245@OG	DC3_74@OP1	25
ARG_54@NH1	DA_12@OP2	21	ARG_249@NH1	DA_72@OP1	16
ARG_54@NH2	DA_12@OP2	23	ARG_249@NH2	DA_72@OP1	21
ALA_84@N	DT_14@OP2	80	THR_257@OG1	DT_8@OP2	96
THR_86@OG1	DA_15@OP2	20	THR_257@N	DT_8@OP2	84
THR_87@OG1	DT_14@OP2	98	GLY_281@N	DA_66@OP1	11
SER_108@OG	DA_58@OP2	14	GLY_281@N	DA_66@OP2	11
ARG_118@NE	DC_57@OP1	12	TYR_283@OH	DA_15@OP1	28
ARG_118@NH2	DA_58@OP2	11	ARG_284@NH1	DA_66@OP2	56
ARG_121@NH1	DC_57@OP1	16	ARG_284@NH2	DT_65@OP1	45
ARG_121@NH1	DC_57@OP2	60	ARG_284@NH2	DA_66@OP2	15
ARG_121@NH2	DC_57@OP1	83	THR_287@OG1	DT_64@OP2	11
ARG_122@NH1	DT_24@OP1	18	HIS_289@N	DT_64@OP2	11
ARG_122@NH2	DT_24@OP1	29	ARG_292@NE	DT_17@OP1	12
THR_131@OG1	DA_15@OP1	24	ARG_292@NH2	DT_17@OP1	10
ARG_154@NH1	DA_6@OP1	10	TRP_315@NE1	DT_17@OP2	18
ARG_159@NH1	DC_7@OP1	97	LYS_316@NZ	DT_18@OP1	21
ARG_159@NH2	DC_7@OP1	30	LYS_317@NZ	DT_18@OP2	57
ARG_159@NH2	DC_7@OP2	27	THR_320@OG1	DT_17@OP2	64
ARG_173@NE	DT_63@OP1	10	ARG_323@NH1	DA_16@OP2	65
ARG_173@NH1	DT_63@OP1	15	ARG_323@NH2	DA_16@OP2	37
ARG_173@NH2	DT_63@OP1	15	ARG_323@NH2	DA_16@OP1	26
ARG_173@NH2	DT_17@OP1	13	TYR_324@OH	DA_16@OP1	84
SER_175@OG	DT_63@OP1	34	-		

Protein	Backbone (S)	% Occurrence	Protein	Backbone (S)	% Occurrence
HIS_243@NE2	DA_72@O3'	11	-		

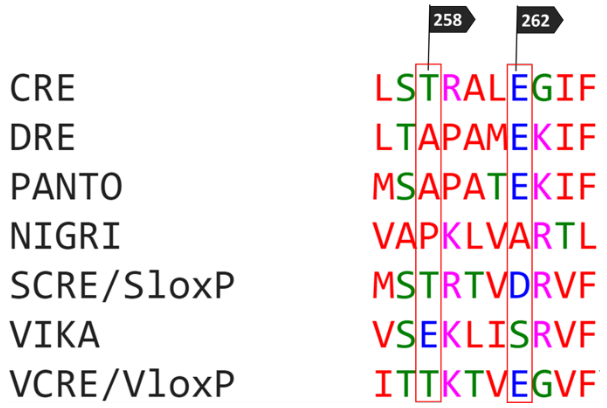

Fig. S4. Sequence alignment of nearest-neighbor amino acids on helix J of naturally occurring Cre-like SSRs.

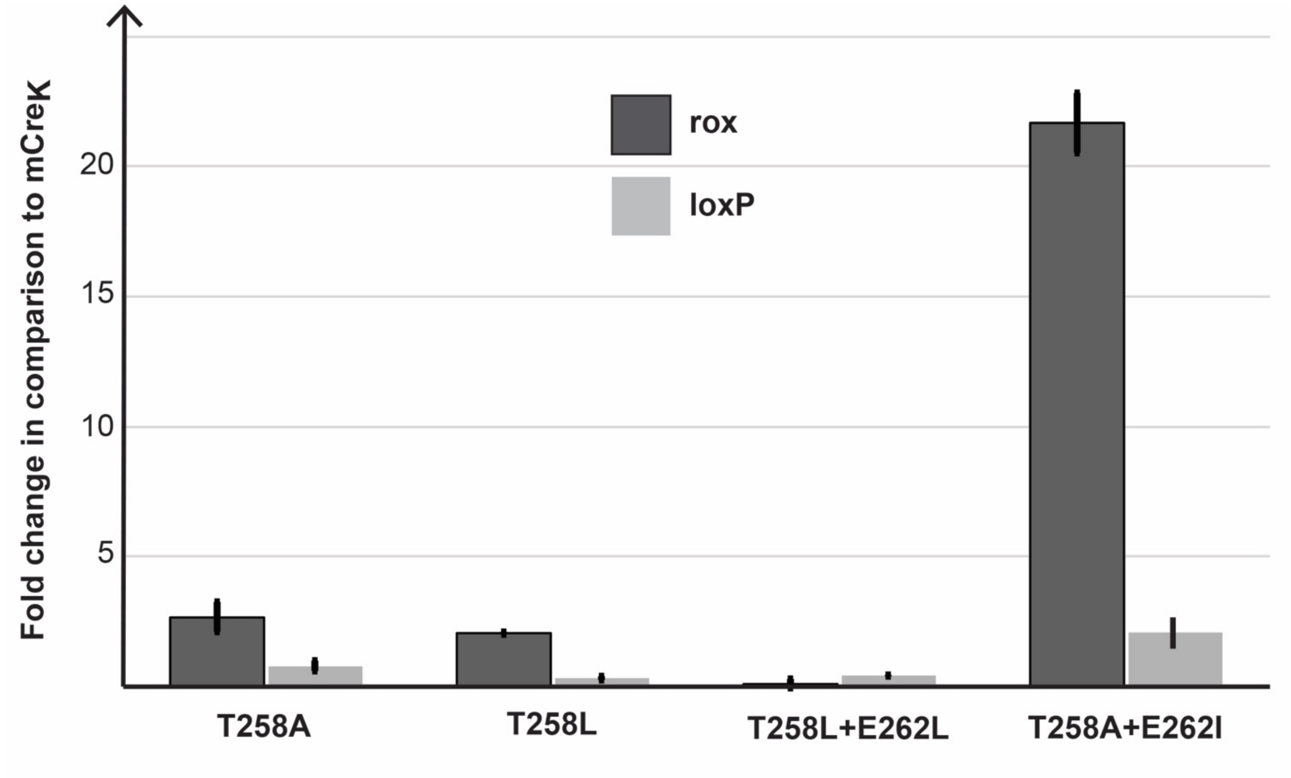

Fig. S5. Recombination activity of indicated mutants in comparison to mCre$_{\kappa}$ on loxP and rox. Fold changes were calculated from band intensities from gels shown in Fig. 7 at 10 µg/ml L-arabinose. Error bars show the standard deviation from three independent experiments.

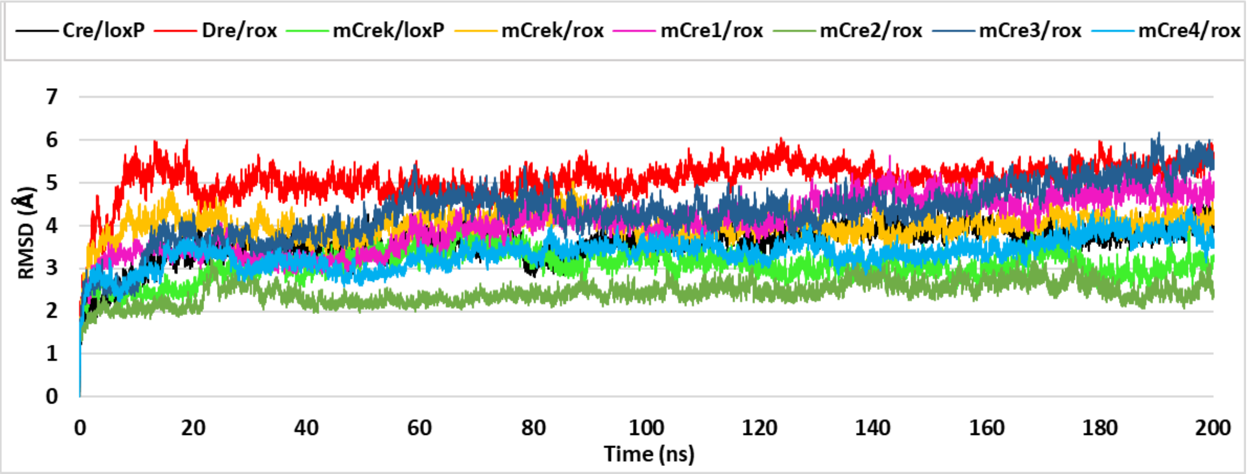

Fig. S6. Root mean square deviation (RMSD) with respect to time for the studied Protein-DNA complexes.

Table S3. Calculated MM-GB/PBSA binding energies (kcal/mol) and corresponding standard deviations for the studied SSRs complexes (for a period of 100 to 200 ns)

**Complexes**	ΔE_{vdW}	ΔE_{ele}	ΔG_{GB}	ΔG_{PB}	$\Delta G_{SA(GB)}$	$\Delta G_{SA(PB)}$	ΔG_{GBSA}	ΔG_{PBSA}
**Cre/loxP**	-523.7 ± 13.5	-28054.3 ± 304.7	28002.3 ± 297.7	27851.9 ± 296.2	-75.5 ± 1.3	-57.6 ± 0.7	-651.3 ± 26.0	-783.8 ± 24.5
**Dre/rox**	-435.2 ± 15.9	-20730.1 ± 272.3	20785.4 ± 271.5	20632.3 ± 270.8	-62.7 ± 1.5	-49.2 ± 0.9	-442.6 ± 36.2	-582.1 ± 33.9
**mCre$_{\kappa}$/loxP**	-504.5 ± 18.0	-27888.9 ± 274.4	27839.1 ± 267.6	27671.9 ± 268.4	-72.9 ± 1.5	-54.9 ± 0.9	-627.4 ± 25.7	-776.5 ± 26.0
**mCre$_{\kappa}$/rox**	-521.7 ± 14.5	-26955.1 ± 366.2	26888.1 ± 359.0	26745.0 ± 363.9	-74.0 ± 1.2	-56.2 ± 0.8	-662.8 ± 24.8	-788.1 ± 24.1
**mCre1/rox**	-494.8 ± 11.5	-27110.2 ± 217.6	27071.3 ± 216.8	26882.6 ± 217.8	-70.7 ± 1.3	-54.6 ± 0.7	-604.4 ± 31.3	-777.1 ± 27.7
**mCre2/rox**	-503.1 ± 13.6	-27153.3 ± 284.6	27117.3 ± 279.2	26957.0 ± 275.8	-71.2 ± 1.3	-55.3 ± 0.8	-610.3 ± 20.2	-754.8 ± 22.9
**mCre3/rox**	-487.2 ± 22.9	-28467.1 ± 388.1	28457.7 ± 376.4	28240.9 ± 390.1	-68.8 ± 2.79	-54.6 ± 1.3	-565.4 ± 42.8	-767.9 ± 27.6
**mCre4/rox**	-529.7 ± 14.5	-28915.5 ± 295.5	28852.1 ± 286.6	28688.8 ± 292.7	-74.9 ± 1.1	-55.8 ± 0.7	-668.1 ± 25.2	-812.2 ± 27.3
